# Supplementary material for: The role of π-blocking hydride ligands in a pressure-induced insulator-to-metal phase transition in SrVO2H
Source: Nat Commun. 2017 Oct 31;8:1217. doi: 10.1038/s41467-017-01301-0 (PMC5663929; doi:10.1038/s41467-017-01301-0)
Supplement: Supplementary file 1 — Supplementary Information [file 41467_2017_1301_MOESM1_ESM.pdf]

Supplementary Information

Supplementary Note 1. Sample dependence of Diffraction and Resistivity measurements

In the experimental component of this work, we have used two different samples of $SrVO_2H$, $SrVO_2H$-A and $SrVO_2H$-B. The structural results reported in Fig. 2 of the main text relate to $SrVO_2H$-A, where a small amount (~14%) of $SrVO_3$ impurity was found to be present. The impurity peaks, identified by comparison to pure $SrVO_3$ (Supplementary Fig. 1), are clearly identifiable in Figure 2a, and do not adversely affect the analysis of the diffraction data. However, initial measurements of the resistivity of sample $SrVO_2H$-A showed only rather weak dependence on pressure and temperature (Supplementary Fig. 5, a,b,c). We therefore synthesized a second sample, $SrVO_2H$-B, which had fewer impurities present, and conducted pressure- and temperature-dependent resistivity experiments. The X-ray data for the two samples are compared in Supplementary Fig. 1. The reproducibility was checked by measuring the resistivity twice using different batches of $SrVO_2H$-B（Fig. 3 in the main text and Supplementary Fig. 4）. The resistivity of $SrVO_2H$-B is substantially higher than that for $SrVO_2H$-A, and the pressure and temperature dependences span 2 orders of magnitude more. Nevertheless, the trend towards a more highly conducting state with increased pressure is apparent in both data sets.

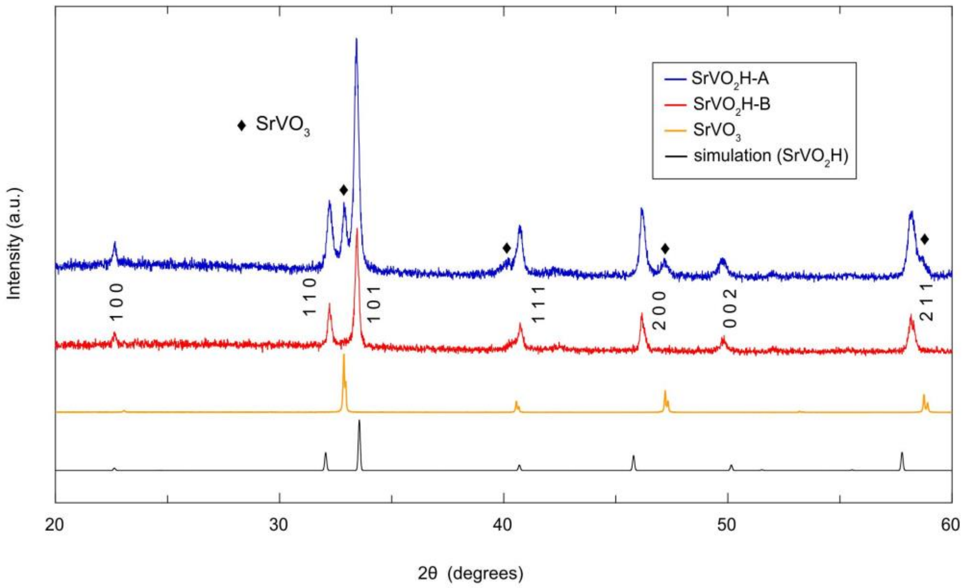

Supplementary Figure 1. XRD patterns of $SrVO_2H$. XRD patterns of $SrVO_2H$-A (blue), $SrVO_2H$-B (red) and $SrVO_3$ (orange) and simulation pattern for $SrVO_2H$ (black). Data were measured on a D8 ADVANCE diffractometer (Bruker AXS) with Cu-Kα radiation. $SrVO_2H$-A and $SrVO_2H$-B were used for resistivity measurements. $SrVO_2H$-A contains unreduced $SrVO_3$ impurity with 14% of molar fraction.

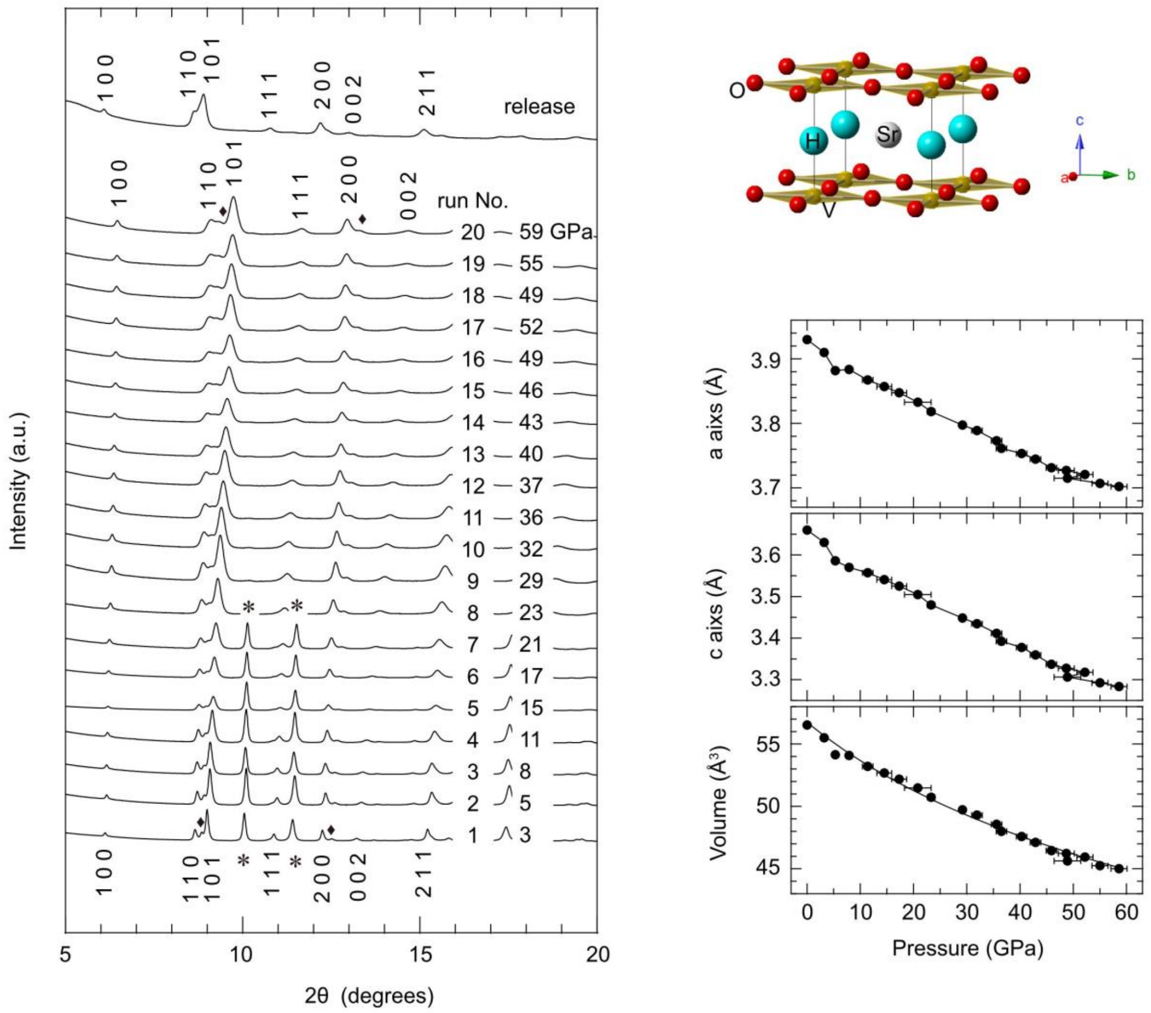

Supplementary Figure 2. High pressure XRD experiments on $SrVO_2H$. (left) Powder synchrotron XRD patterns of sample $SrVO_2H$-A under pressure at room temperature (λ = 0.4173 Å). All patterns could be indexed on the basis of the tetragonal unit cell. The symbols ♦ and ∗ correspond to $SrVO_3$ and rhenium from the gasket, respectively. (right) Pressure dependence of the lattice parameters and the volume. The solid line in the volume plot represents the Birch-Murnaghan fitting with bulk modulus K of 160(5) GPa.

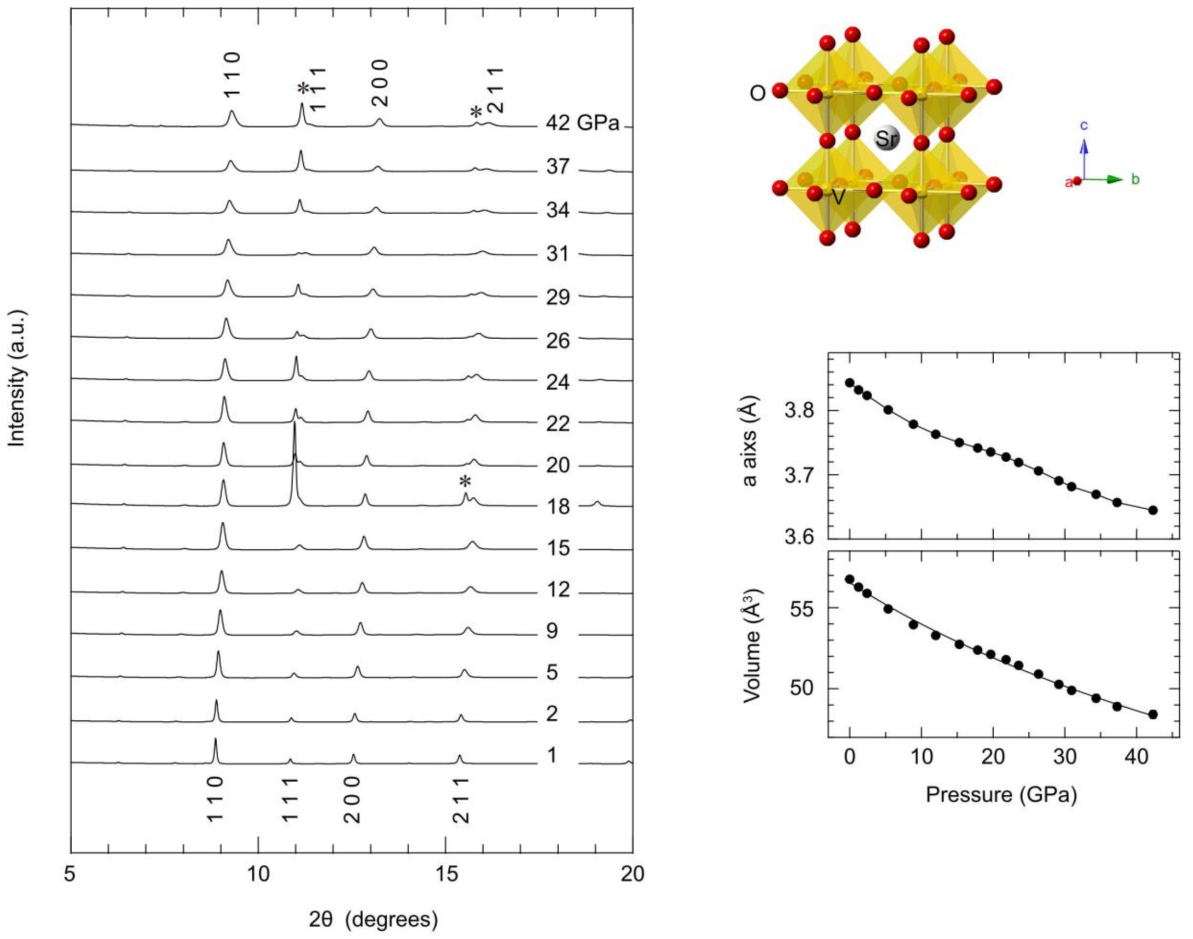

Supplementary Figure 3. High pressure XRD experiments on $SrVO_3$. (left) Powder synchrotron XRD patterns under pressure at room temperature (λ = 0.4186 Å). The patterns can be indexed using a cubic cell. ∗ corresponds to tungsten-rhenium alloy from gasket. (right) Pressure dependence of the lattice parameters and the volume. Solid line in volume represents Birch-Murnaghan fitting with bulk modulus *K* of 196(4) GPa.

Supplementary Note 2. Linearized Birch-Murnaghan fitting and zero-pressure linear compressibility β

Linearized Birch-Murnaghnan equation of state:

$$P = \frac{3}{2}K_{l0}\left[\left(\frac{l_0}{l}\right)^7 - \left(\frac{l_0}{l}\right)^5\right] \times \left\{1 - \frac{3}{4}(4 - K_l') \times \left[\left(\frac{l_0}{l}\right)^2 - 1\right]\right\} \quad (1)$$

where l, K_{l0} and K_l' represent each axis (a, b, and c), the axial modulus, and the pressure derivative of the l-axis. K_l' was fixed to 4 in all the fitting.

The zero-pressure linear compressibility β_l is determined as:

$$\beta_l = -\frac{1}{l}\left(\frac{\partial l}{\partial P}\right)_T = \frac{1}{3K_{l0}} \quad (2)$$

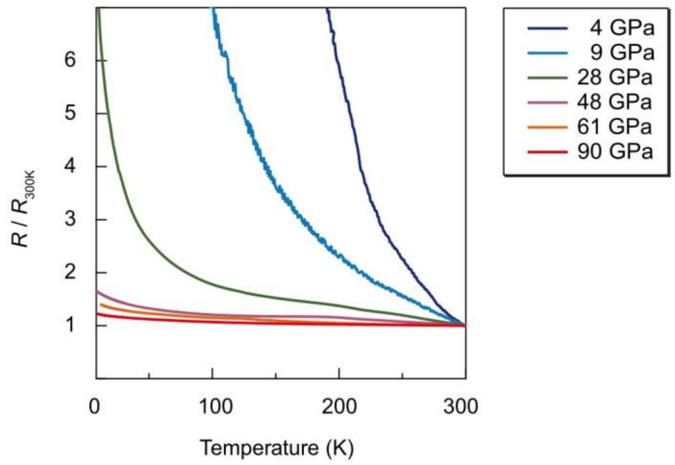

Supplementary Figure 4. Normalized resistivity *R/R*$_{300K}$ under pressure. This data are from experiments of Fig. 3. The resistance becomes essentially independent of temperature above 48 GPa, indicating an insulator-to-metal transition.

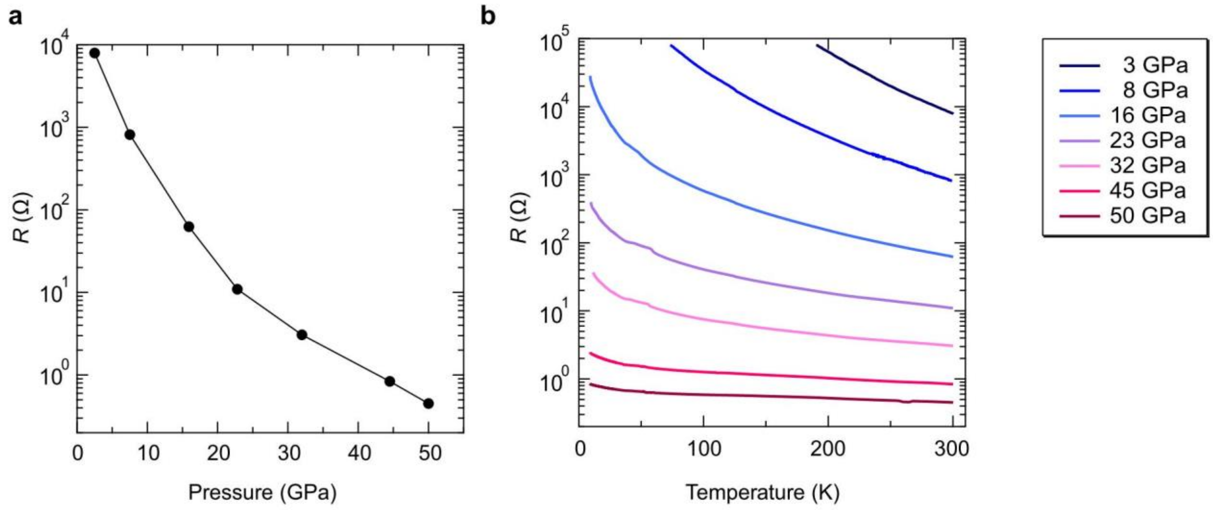

Supplementary Figure 5. Electrical resistivity for $SrVO_2H$-B under pressure. Pressure dependence of resistivity R at 300 K (**a**), Temperature dependence of resistance (**b**). This measurement was a different run from that of Fig. 3. Note that this measurement reproduces the results shown in Fig. 3, but represents an independent run.

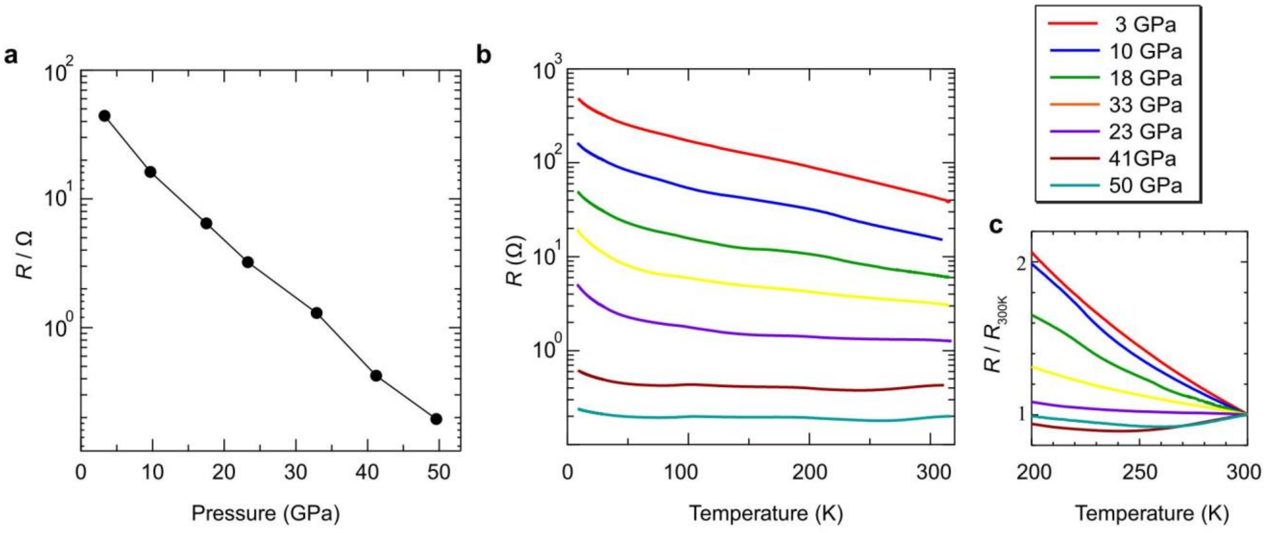

Supplementary Figure 6. Electrical resistivity for $SrVO_2H$-A under pressure. Pressure dependence of resistivity R at 300 K (**a**), Temperature dependence of resistance (**b**), normalized resistivity R/R_{300K} (**c**).

Supplementary Note 3. Anomaly in the lattice parameters

The pressure-dependence of lattice parameters is shown in Supplementary Table 1 (the corresponding XRD patterns are shown in Figure 2S). Supplementary Fig. 7 shows the pressure plotted against volume and rotation of screw. When the screw is rotated, the distance between the diamond anvils become closer and the volume of sample space decreases. So pressure generally increases when the screw is rotated. The anomaly referred to in the text corresponds run No. 18 in Supplementary Table 1 (and red circles in Supplementary Fig. 7) where the pressure drops from 52 GPa to 49 GPa. This clearly shows that the sample has become much denser in a discontinuous manner, consistent with a volume reduction at an insulator-to-metal transition. The pressure was measured using two different ruby chips and both the chips indicated a decrease of pressure. Similar behavior has been observed in Sr_2PdO_3 at a pressure-induced structural transition (*Inorg. Chem.,* **50**, 11787−11794 (2011)).

Supplementary Table 1. Lattice parameters of $SrVO_2H$

Run No.	Rotation of screw (°)	Pressure (GPa)	*a* axis (Å)	*c* axis (Å)
0	–	0	3.93	3.66
1	0	3.2	3.90995	3.6302
2	30	5.3	3.88185	3.58585
3	45	7.9	3.88353	3.56996
4	60	11.4	3.86731	3.55725
5	75	14.5	3.85712	3.54061
6	105	17.3	3.84747	3.52509
7	120	20.8	3.83271	3.50477
8	135	23.3	3.8183	3.47979
9	165	29.2	3.79735	3.44848
10	180	31.9	3.78884	3.4346
11	195	35.6	3.77309	3.4114
12	210	36.5	3.76138	3.39232
13	225	40.3	3.7533	3.37758
14	240	42.9	3.74469	3.35957
15	255	45.9	3.73127	3.33716
16	270	48.7	3.72699	3.32747
17	285	52.2	3.72088	3.31771
18	300	48.9	3.71498	3.30642
19	315	55	3.70701	3.29268
20	330	58.6	3.70212	3.28343

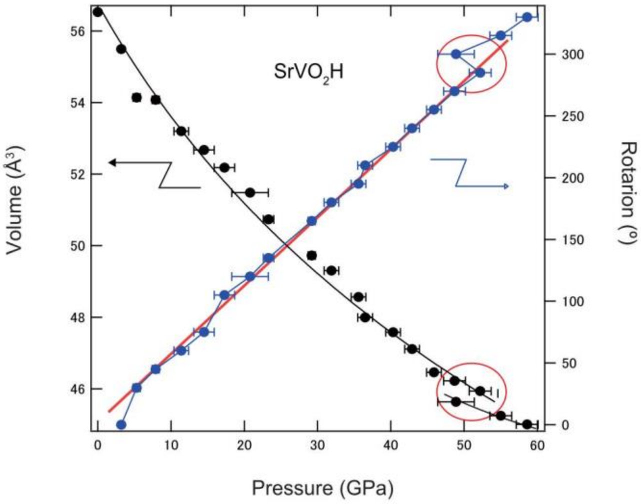

Supplementary Figure 7. Pressure dependence of Volume and rotation of screw. Anomaly at ~ 50 GPa (red circles) implies a phase transition. Red line represent a linear fitting curve between 5 GPa to 52 GPa.

Supplementary Note 4. Computational details

The $\sqrt{2} \times \sqrt{2} \times 2$ expansion of the unit cell used in all computations is shown in Supplementary Fig. 8.

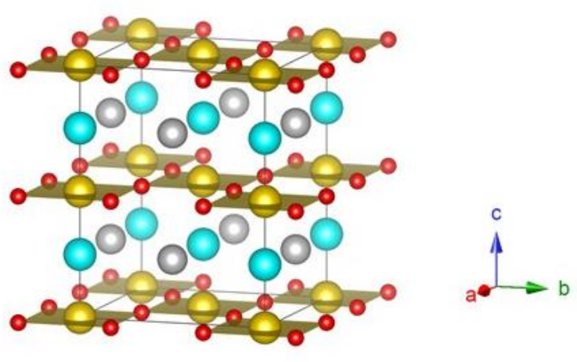

Supplementary Figure 8. The $\sqrt{2} \times \sqrt{2} \times 2$ expansion of the unit cell used to explore the magnetic configurations.

Optimized lattice parameters. The optimized cell parameters for a $\sqrt{2} \times \sqrt{2} \times 2$ expansion of the primitive unit cell are collected in Supplementary Table 2 for a range of U_{eff} values from 0.0 through to 5.0 eV. In all cases the optimizations correspond to the *G*-type antiferromagnetic configuration that proves to be the most stable coupling configuration. The spin densities on the vanadium centers confirm the expected increased localization of the valence electrons as U_{eff} increases, the U_{eff} = 5.0 eV limit of ρ(V) = 1.88 being typical of a formally d^2 ion in an oxide lattice. The agreement between X-ray and optimized geometries is generally good (within 3%).

Supplementary Table 2. Optimized structural parameters in the *G*-type antiferromagnetic configuration of $SrVO_2H$

U_{eff} / eV	Total Energy /eV	$\boldsymbol{a}$ / Å	$\boldsymbol{c}$ / Å	Mulliken Spin Density
0.0	–135.43	3.93	3.69	±1.39
0.5	–133.90	3.95	3.69	±1.56
1.0	–132.52	3.96	3.69	±1.64
2.0	–129.92	3.98	3.69	±1.73
3.0	–127.51	4.00	3.70	±1.80
4.0	–125.24	4.01	3.71	±1.84
5.0	–123.09	4.03	3.73	±1.88
Exp.	-	3.93	3.67	-

Exchange coupling pathways. The nature and strength of the magnetic coupling between the vanadium centers has been discussed previously by both Wei *et al*[1]. and by Liu *et al.*[2]. The observed *G*-type antiferromagnetism indicates the presence of strong intra-layer coupling *via* the bridging oxides due efficient superexchange between the singly occupied $3d_{xz}$ and $3d_{yz}$ orbitals, mediated by the oxide $2p$ orbitals. The origin of the strong inter-layer exchange is less obvious, as both $3d_{z^2}$ orbitals are formally vacant. In order to evaluate the strength of exchange along these four distinct pathways we map the DFT-computed energies of the *G*-, *C*-, *A*-, *X*- and *F*- configurations (Supplementary Fig. 9) onto the diagonal elements of the Heisenberg Spin Hamiltonian computed in the basis of uncoupled states:

$$\hat{H}_{Heisenberg} = 2J_{ij} \sum_{j>i} \hat{\boldsymbol{S}}_i \cdot \hat{\boldsymbol{S}}_j \tag{3}$$

J_{ij} is the effective exchange coupling constant between atoms i and j with $\hat{S}_i = \hat{S}_j = 1$. In the $\sqrt{2} \times \sqrt{2} \times 2$ supercell defined in Supplementary Fig. 8 we can identify four symmetry-distinct exchange coupling paths, $J_{1\text{-}4}$: J_1 corresponds to the nearest neighbor in-plane V-O-V coupling while J_2, in contrast, is the nearest neighbor inter-planar V-H-V coupling. A strontium cation lies directly along the line of the J_3 pathway while there is no bridging ion at all directly along the J_4 pathway. Our computed values of J_1 = 20.0 meV; J_2 = 4.5 meV; J_3 = 0.9 meV; J_4 = 0.2 meV (U_{eff} = 2.0 eV) confirm that the interactions J_3 and J_4 are small and can safely be neglected in this analysis. Like Wei and Liu, we conclude that the experimentally observed *G*-type antiferromagnet[3] is the most stable configuration, with stronger coupling in the *ab*-plane (V–O–V, J_1 = 20.0 meV) than along the *c*-axis (V–H–V, J_2 = 4.5 meV). The large antiferromagnetic J_1 is a result of strong superexchange between the singly occupied $d_{xz/yz}$ orbitals, mediated by O p_z, and is to be anticipated based on the Goodenough-Kanamori rules[4]. The rather large value for J_2, the inter-planar exchange coupling, is in contrast somewhat unexpected given that the V-H-V coupling pathway *via* the metal d_{z^2} orbitals corresponds to a formally 'empty-empty' interaction, which usually leads to only weak coupling. Nevertheless, our value of J_2 is consistent with those reported previously by both Wei *et. al* and by Liu *et. al*. Wei *et al*. have argued for direct through-space interactions between the two vanadium centers along *c*, although the V–V separation along the *c*-axis is greater than the bonding distance in, *e.g.*, VO_2, suggesting that such overlap should be rather small. An alternative explanation comes from the significant covalent

character of the V–H bonds. The Goodenough-Kanamori rules predict an antiferromagnetic contribution to the coupling in this case because charge transfer from H $1s$ to the vacant V d_{z^2} orbitals is most efficient when the two electrons in the hydride $1s$ orbital are transferred to different metal centers. In a ferromagnetic arrangement, in contrast, only one of the H $1s$ electrons can undergo a virtual transfer to the metal $3d_{z^2}$ (Supplementary Fig. 10). Similar factors lead to the high ordering temperature in $LaSrCoO_3H_{0.7}$ [5].

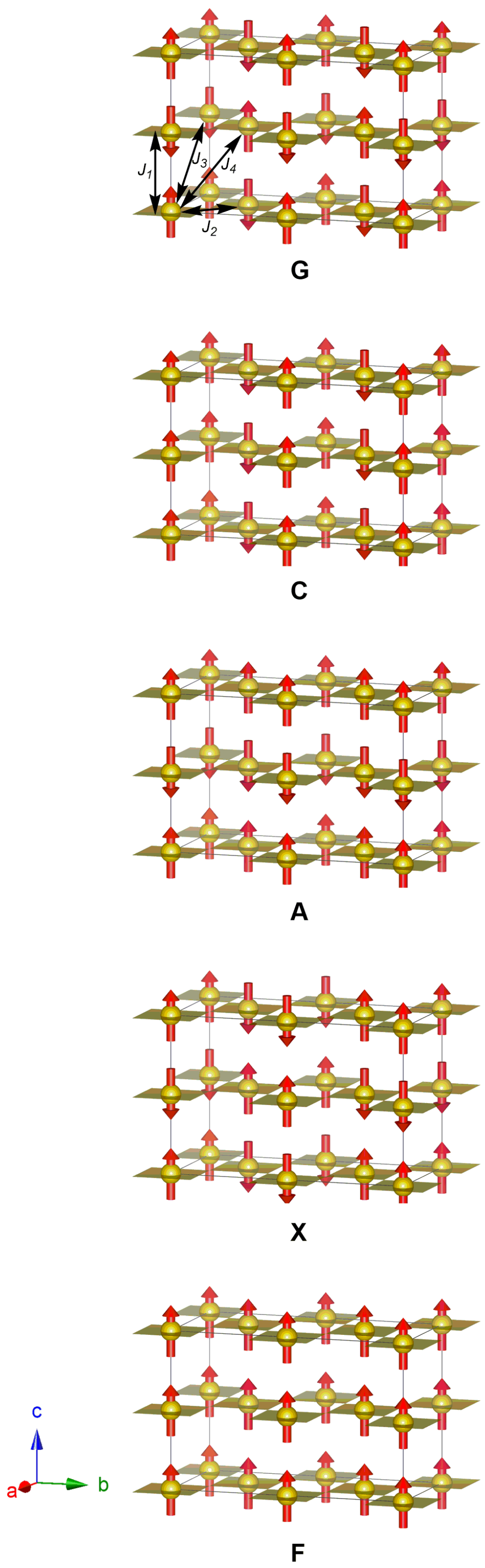

Supplementary Figure 9. *G*-, *C*-, *A*-, *X*- and *F*- configurations.

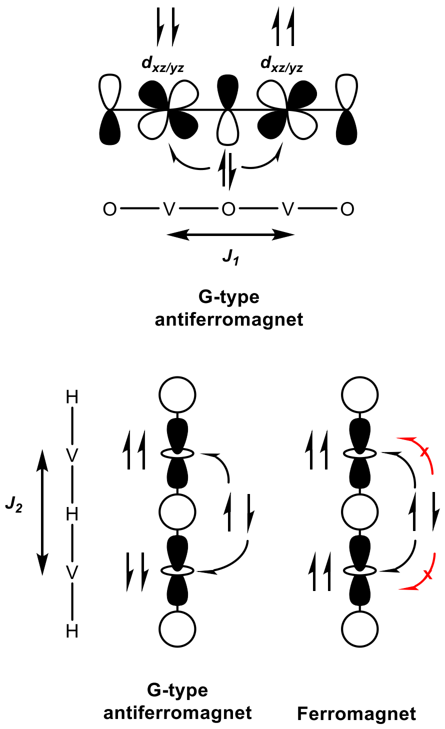

Supplementary Figure 10. Schematic picture of exchange coupling pathways for the intra-plane and inter-plane antiferromagnetism.

Pressure dependence of the band structure. In the main text, we showed that application of pressure causes a contraction along both *c* and *a*, and that this leads to a transition to a metallic state above 50 GPa. In order to separate out the roles of compression along *c* and compression along *a* in forcing this transition, we have performed a series of calculations where the *c* and *a* lattice parameters are constrained at different values that fall between the high- and low-pressure limits (Supplementary Fig. 11). The important point to emerge from this figure is that compression along *c* alone does not induce a transition to a metallic state, even at the shortest distances. In contrast, compression along *a* does cause a metallic state to form, even when *c* remains at its ambient pressure value. From this, we conclude that compression along *a* is more important in the phase transition, even though it is smaller in absolute terms than compression along *c*.

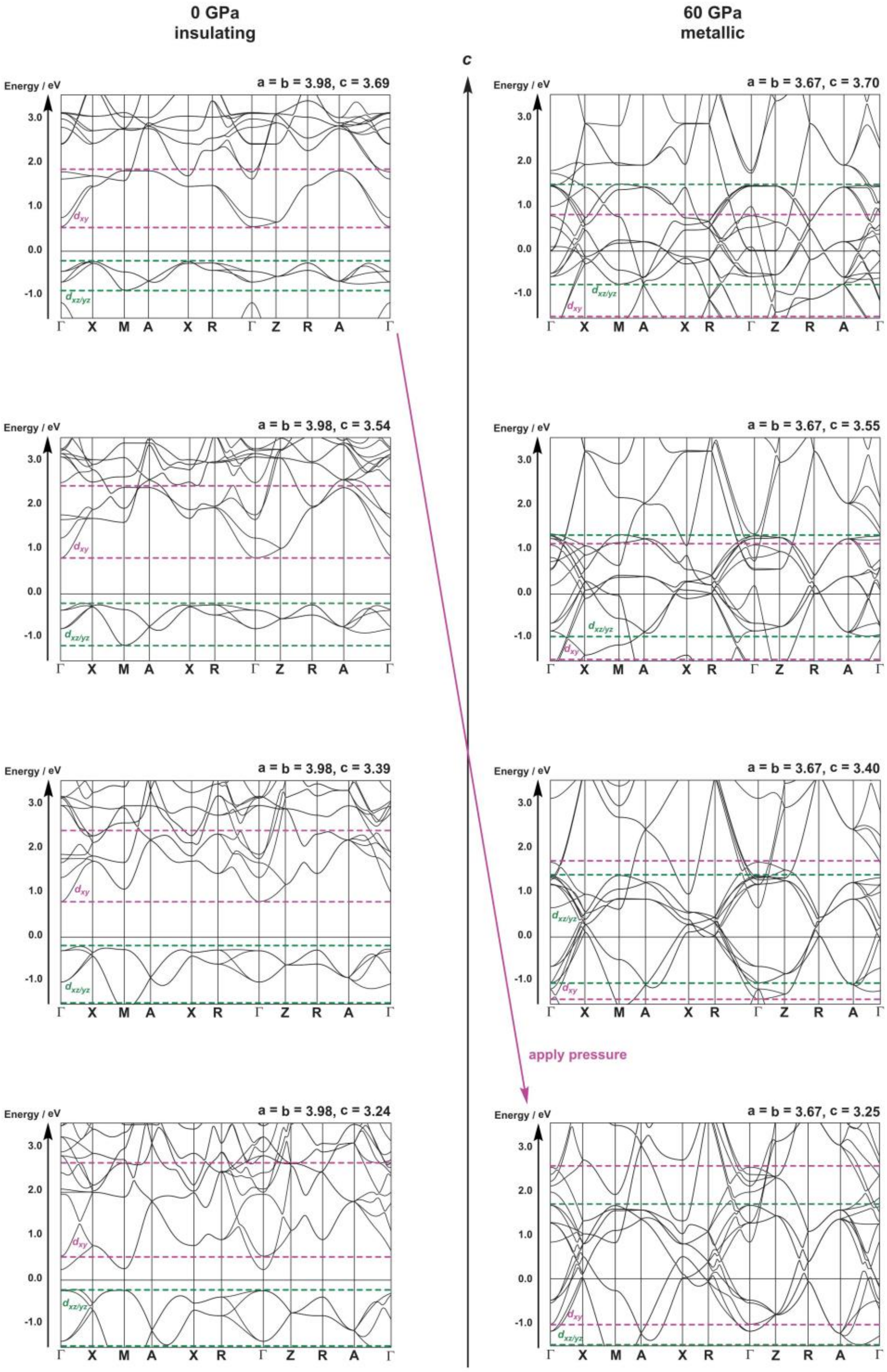

Supplementary Figure 11. Variation of band structure as a function of lattice parameters *c* and *a* (in Å).

Influence of choice of methodology on the electronic structure analysis. We have noted above that the choice of Hubbard U_{eff}, makes subtle differences to the computed ground-state electronic structure at ambient pressure. Increasing U_{eff}, increases the spin density on the vanadium centers and increases the lattice parameters, a and c. The density of states plots as a function of pressure for U_{eff} = 0, 2.0 and 4.0 eV in Supplementary Fig. 12 show that increasing U_{eff} increases the band gap at ambient pressure, and therefore pushes the insulator-to-metal transition to higher pressures. Thus even at 60 GPa, the U_{eff} = 4.0 eV calculations predict an insulating state with a finite band gap. An alternative strategy that does not require a parameter choice is to use a hybrid functional such as HSE06, which in principle alleviates some of the self-interaction error that compromises the accuracy of the band gap in GGA functionals such as PBE. The density of states computed with the HSE06 functional are rather similar to those obtained with U_{eff} = 4.0 eV across the entire pressure range, and indeed the system remains insulating even at 60 GPa. Despite the absolute differences in these plots, it is clear that the effect of pressure is qualitatively the same in all cases: the dominant feature is the greater dispersion in the d_{xy} conduction band. Thus while the exact value of the critical pressure is strongly dependent on methodology, the conclusions regarding the underlying mechanism are not.

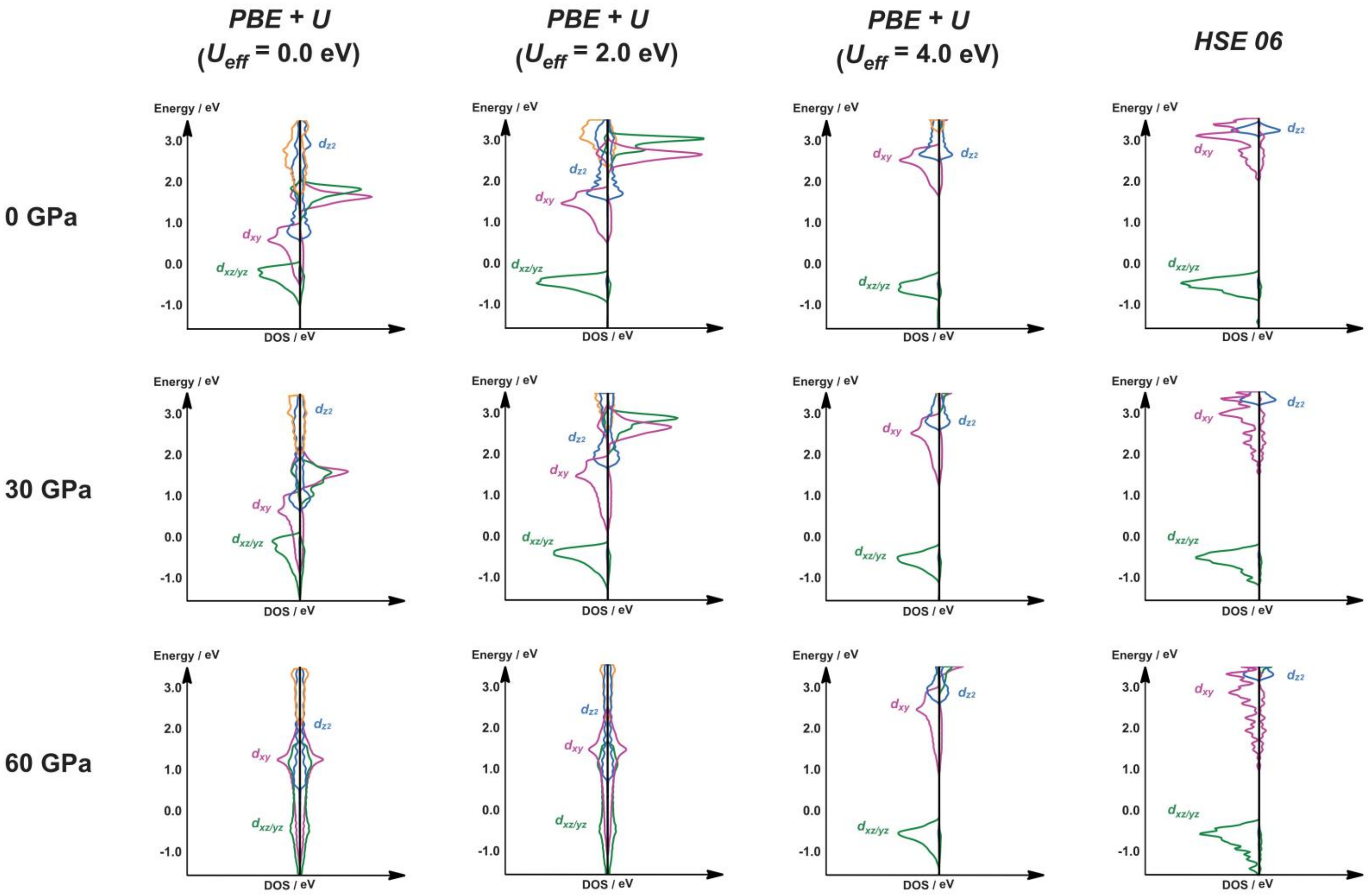

Supplementary Figure 12. Pressure-dependent density of states computed using different computational protocols.

The calculation of the transmission spectrum for the metallic state necessitated a change in methodology, from a plane-wave basis as used in VASP to a localized basis set in AtomwiseToolKit (ATK). Before analyzing the transmission, we therefore need to establish that the two codes are giving a quantitatively similar description of the electronic structure. The total DOS for the system computed with VASP and ATK (for the 60GPa structure optimized with the former) are shown in Supplementary Figure 13, and are almost identical. We are therefore confident that the transmission spectrum from ATK can be mapped onto the projected DOS computed using VASP.

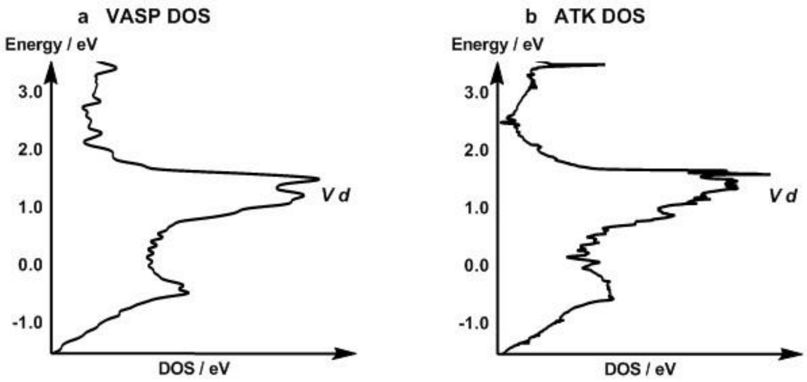

Supplementary Figure 13. Comparison of the density of states obtained from VASP and ATK (both with the PBE functional and U_{eff} = 2.0 eV).

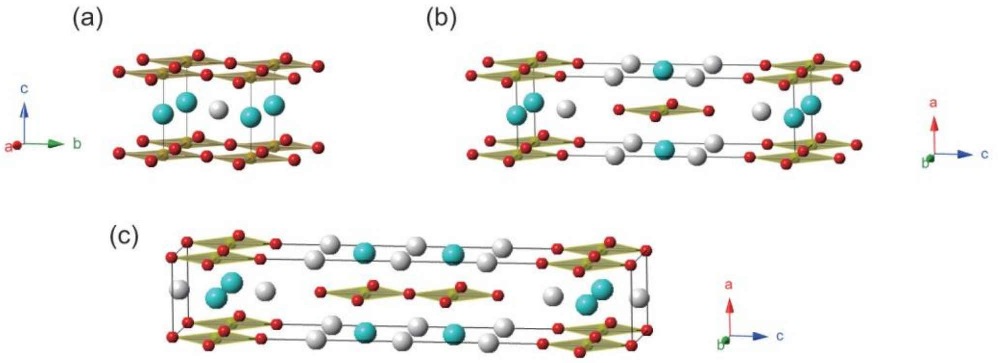

Supplementary Figure 14. Crystal structures of $Sr_{n+1}V_nO_{2n+1}H_n$ (n = 1, 2, ∞). a, $SrVO_2H$ ($n = \infty$), **b,** Sr_2VO_3H ($n = 1$), **c,** $Sr_3V_2O_5H_2$ ($n = 2$). White, yellow, red, and sky blue spheres respectively denote Sr, V, O, H atoms.

Supplementary references

1 Wei, Y., Gui, H., Li, X., Zhao, Z., Zhao, Y. H. & Xie, W. The effect of hydrogen ordering on the electronic and magnetic properties of the strontium vanadium oxyhydride. *J. Phys. Condens. Matter* **27,** 206001 (2015).

2 Liu, K., Hou, Y. S., Gong, X. G. & Xiang, H. J. Orbital delocalization and enhancement of magnetic interactions in perovskite oxyhydrides. *Sci. Rep.* **6,** 19653 (2016).

3 Denis Romero, F., Leach, A., Moller, J. S., Foronda, F., Blundell, S. & Hayward, M. A. Strontium vanadium oxide-hydrides: 'square-planar' two-electron phases. *Angew. Chem. Int. Ed.* **53,** 7556–7559 (2014).

4 Goodenough, J. B. Magnetism and the Chemical Bond; Interscience: New York; London, 1963.

5 Bridges, C. A., Darling, G. R., Hayward, M. A. & Rosseinsky, M. J. Electronic structure, magnetic ordering, and formation pathway of the transition metal oxide hydride $LaSrCoO_3H_{0.7}$. *J. Am. Chem. Soc.* **127,** 5996–6011 (2005).
